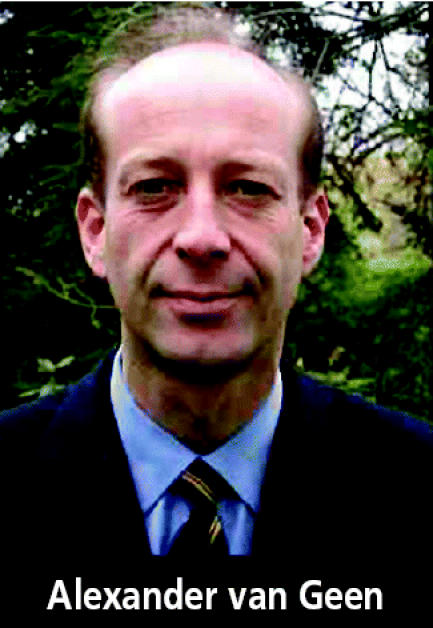# Guest Editorial: Reducing Arsenic Exposure from Drinking Water: Different Settings Call for Different Approaches

**DOI:** 10.1289/ehp.113-a360

**Published:** 2005-06

**Authors:** Joseph H. Graziano, Alexander van Geen

**Affiliations:** Columbia University, New York, New York, E-mail: jg24@columbia.edu; avangeen@ldeo.columbia.edu

On 1 January 2006, a new U.S. drinking water standard of 10 μg arsenic/L will come into effect [U.S. Environmental Protection Agency (EPA) 2001a). We strongly support the U.S. EPA’s decision to lower the allowable limit of As in drinking water from 50 μg/L to 10 μg/L because it promises to reduce the risk of an array of adverse health outcomes attributable to As exposure, notably various cancers and cardiovascular and neurologic diseases.

Throughout the United States, but particularly in the northeastern and southwestern states, where drinking water sources are most likely to exceed the 10 μg/L limit, public agencies responsible for water quality are preparing for the arrival of the new standard in a variety of ways. In 2001, the U.S. EPA estimated that the arsenic content of water provided by roughly 5% of U.S. community water supplies exceeded 10 μg/L (U.S. EPA 2001b); in these cases, the introduction of water-treatment facilities will be required to bring systems into compliance. Although this will be expensive, the ever-increasing evidence that waterborne arsenic is a menace to public health—including new findings that it impacts children’s intellectual functioning (Wasserman et al. 2004)—warrants the cost.

A significant segment of the U.S. population at risk, however, relies on individual household wells for their drinking water. Groundwater studies conducted by the U.S. Geological Survey (Focazio et al. 1999) imply that nearly 8% of domestic wells exceed the new As standard. Here, the responsibility for water treatment lies with the homeowner. Simple over-the-counter filtration systems are not effective for removing As from tap water. Rather, more elaborate technologies costing several thousand dollars (e.g., reverse osmosis systems) are required. For those who can afford it, the cost of installing such systems to protect family health is small, but for those who are economically disadvantaged, a water treatment system to remove As (and other potentially harmful elements) may not be a high priority. To help alleviate the situation, testing of household water for As should become part of the building-inspection process that preceeds the sale of a home, allowing for the cost for water treatment to be factored into the transaction.

In comparison to the situation in Bangladesh and other developing nations, the U.S. problem is small and readily solvable. Although estimates vary, perhaps as many 100 million rural inhabitants of Bangladesh and other affected South Asian countries drink untreated well water with As concentrations that can exceed the Bangladesh standard of 50 μg/L by more than an order of magnitude. A single visit to a severely affected region of Bangladesh can be a life-altering experience, as the skin lesions associated with the consumption of As-contaminated water are evident, even in young children. When one realizes that skin lesions are but a visible manifestation of a wider syndrome that damages multiple internal organ systems, the magnitude of the arsenic problem becomes even more unsettling.

The extent of the problem, coupled with the relative economic plight of the country, drives home the need for a more significant response by developed nations—and the donor community—to assist Bangladesh as it works toward achieving a safe water supply. Despite continued efforts by the government of Bangladesh, scientists, industry, and other governmental and nongovernmental organizations, large-scale removal of arsenic from groundwater or human pathogens from surface water appears to be an exceedingly difficult objective to achieve in the near future.

A temporary solution appears to be at hand in thousands of affected villages, but residents are often not aware of it. Deeper aquifers are typically low in As. Over the past several years, the World Bank–sponsored Bangladesh Arsenic Mitigation Water Supply Project (BAMWSP) has conducted a massive field-testing campaign for arsenic of over 5 million wells across the most affected half of the country (BAMWSP 2005). By and large, these results have been accurate and probably already have led many households to switch from their As-contaminated well to a neighboring low-As well (van Geen et al. 2002). The testing campaign, however, did not address the needs of the many households that could not switch to a safe well because of geographic or social constraints.

The BAMWSP data could also be useful by guiding the installation of community wells to those deeper aquifers that are low in As. In collaboration with scientists from Bangladesh, research conducted by a number of international groups has shown that extraction of drinking water from such aquifers (but not large-volume pumping for irrigation water, which could lead to contamination of the deeper aquifers) is feasible and likely to be sustainable in a majority of villages in Bangladesh [British Geological Survey/Department of Public Health Engineering (BGS/DPHE) 2001; Zheng et al., in press]. The use of community wells that tap these deeper aquifers has been extensive in 50 villages of Araihazar upazila, where health, Earth, and social scientists of Columbia University have been conducting basic research with support from the Superfund Basic Research Program (van Geen et al. 2003).

The valuable BAMWSP arsenic data, which have been compiled with information about well location and depth, should be used in a concerted effort to target aquifers for the installation of community wells across a larger portion of Bangladesh. Although coupling the installation of these community wells to complex piped-water supply systems, as currently favored by the World Bank, should be a longer-term goal, it may slow the process in the short term.

In the significant number of villages where the BAMWSP data do not unambiguously identify a safe depth, exploratory drilling will be needed (Gelman et al. 2004; van Geen et al. 2004). A team supported by the Earth Institute at Columbia University is piloting a cell phone–based system to provide access to the BAMWSP database from any village in Bangladesh and to update the database as new wells are installed. This approach will allow communities to determine the local depth of low-As aquifers and empower them to make an informed decision concerning the eventual placement of a safe community well. All who are involved in As mitigation should make available and advertise, at the village level, local testing for As.

Of the 6,000 wells within a 25-km^2^ area that we tested in 2000–2001, roughly 1,000 had been replaced privately by 2004, partly in response to the previous test results (van Geen et al. 2005); this phenomenon is apparently very widespread. Sadly, these new wells had been installed blindly, and the groundwater pumped from half of the new wells still contained > 50 μg/L As. The spatial variability of As concentrations in Bangladesh ground-water complicates the prediction of the As content of water from a particular well but also provides an opportunity for mitigation in that safe aquifers can be targeted to provide the vast majority of households access to safe water.

## Figures and Tables

**Figure:**
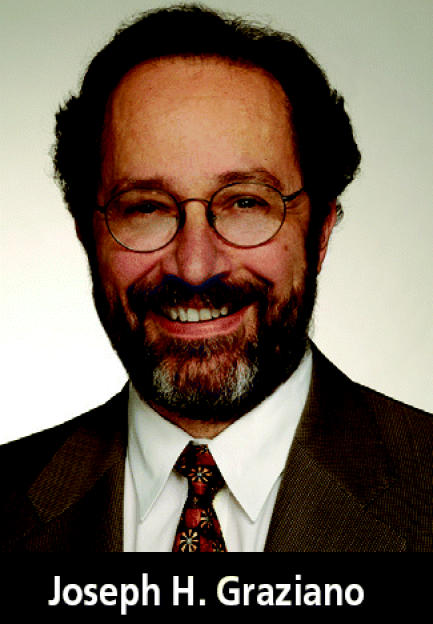


**Figure:**